# A Possible Association Between Executive Dysfunction and Frailty in Patients With Neurocognitive Disorders

**DOI:** 10.3389/fpsyg.2020.554307

**Published:** 2020-11-11

**Authors:** Massimo Bartoli, Sara Palermo, Giuseppina Elena Cipriani, Martina Amanzio

**Affiliations:** ^1^Department of Psychology, University of Turin, Turin, Italy; ^2^European Innovation Partnership on Active and Healthy Ageing, Brussels, Belgium

**Keywords:** frailty, mild cognitive impairment, neurocognitive disorders, Alzheimer’s disease, Parkinson’s disease, executive functions, mini-review

## Abstract

Frailty is an age-related dynamic status, characterized by a reduced resistance to stressors due to the cumulative decline of multiple physiological systems. Several researches have highlighted a relationship between physical frailty and cognitive decline; however, the role of specific cognitive domains has not been deeply clarified yet. Current studies have hypothesized that physical frailty and neuropsychological deficits may share systemic inflammation and increased oxidative stress in different neurodegenerative disorders, such as Alzheimer’s and Parkinson’s disease. However, the role of the executive dysfunction should be investigated in a more detailed way using a multidimensional approach. With this aim, we conducted a review of the literature on the few experimental articles published to discuss the existence of a relationship between frailty and cognitive impairment in neurocognitive disorders, particularly focusing on the domain of executive dysfunction. The data suggest that physical frailty and cognitive decline, especially executive dysfunction, are two aspects strongly linked in mild and major neurocognitive disorders due to Alzheimer’s and Parkinson’s disease. In light of this, a new framework linking aging, cognitive decline, and neurodegenerative diseases is needed. In order to analyze the effects that aging processes have on neural decline and neurocognitive disease, and to identify relevant groups of users and patients, future longitudinal studies should adopt a multidimensional approach, in the field of primary prevention and in the continuum from mild to major neurocognitive disorder.

## Introduction

Frailty is a complex and heterogeneous clinical status described as the loss of harmonic interactions among various dimensions, such as biological, genetic, functional, psychological, cognitive, and social domains (Pilotto et al., 2020), that lead to homeostatic instability. Although the relationship between this issue and poor outcomes has been highlighted, currently there is no gold standard on how to define measure and diagnose frailty (Richards et al., 2018).

**FIGURE 1:**
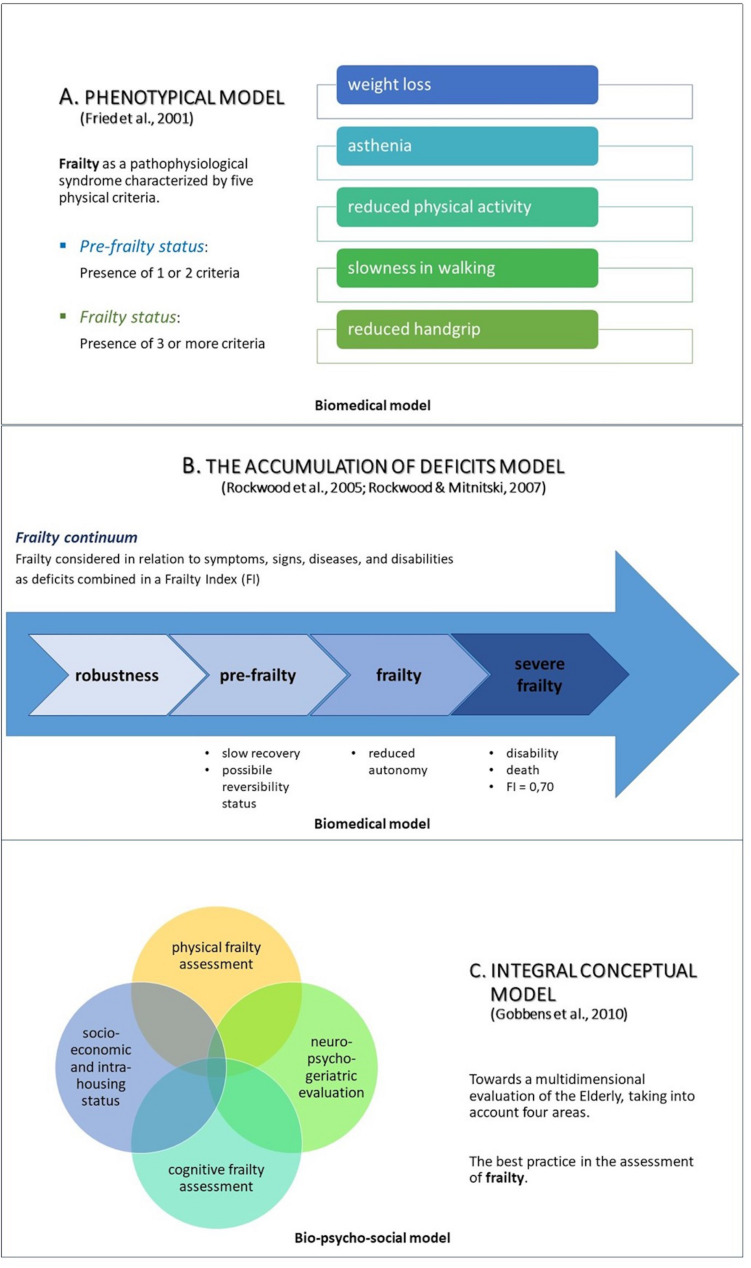
The three main approaches to study frailty: the phenotypical model (Fried et al., 2001; **A**), the accumulation of deficits model (Rockwood et al., 2005; Rockwood and Mitnitski, 2007b; **B**), and the integral conceptual model, based on a bio-psycho-social approach (Gobbens et al., 2010; **C**).

Nowadays, there are at least three main models to study frailty in aging subjects (Figure 1): the phenotypic model (Fried et al., 2001), the deficit accumulation model (Rockwood et al., 2005; Rockwood and Mitnitski, 2007b), and the bio-psycho-social model (Gobbens et al., 2010); the first two characterize the biomedical approach.

The biomedical approach highlights how a reduction in the ability to preserve homeostasis from a physiological point of view, and to respond to environmental changes appropriately, implies a loss of functional autonomy (Xue, 2011).

The phenotypic model (Fried et al., 2001) considers frailty in terms of a physiopathological syndrome composed of five physical determinants: slowness in walking, a decrease in hand-grip-strength, unintentional weight loss, low physical activity, and asthenia. The presence of one or two criteria identifies a pre-frailty status; instead, the presence of three or more, a frailty condition (see Figure 1A).

The deficit accumulation model (Rockwood et al., 2005; Rockwood and Mitnitski, 2007b), may be interpreted in line with a Frailty Index (FI) characterized by age-related deficits, which configure an augmented vulnerability resulting from age-related decline across several body organs and physiological systems. Considering this model of clinical frailty syndrome, the higher the FI, the frailer the individual (see Figure 1B).

Although Rockwood’s model allows for a more extensive evaluation compared to Fried’s one, also demonstrating greater sensitivity in predicting poor outcomes (Rockwood et al., 2007a), it did not fully take into account the psycho-social aspects that may affect the development of frailty.

Over time, the biomedical approach has been criticized (Canevelli et al., 2015) for different reasons: (1) frailty assessment was carried out above all by adopting Fried’s criteria (Fried et al., 2001), as they focused mainly on physical frailty; (2) the majority of these studies evaluated the global cognitive functioning only through the Mini Mental State Examination (MMSE: Folstein et al., 1975), lacking of a full neuropsychological screening; (3) most of the participants were community-dwelling elderly people, compromising the applicability of the results to different types of patients, such as those with neurodegenerative disorders. Therefore, a new concept of frailty has emerged in relation to its applicability in clinical practice. According to this view, frailty can be interpreted as an integrated and multidimensional condition in which multiples domains (such as biological, functional, psychological, and social dimension) interact together, determining and characterizing a frailty status. The above led to the development of the third model, represented by the bio-psycho-social paradigm (Gobbens et al., 2010). Since the interaction of the different “dimensions” is likely to be the basis of the bio-psycho-social and clinical complexity of the frail elderly, multidimensional evaluation is the most suitable choice for frailty detection; it allows to explore not only the physical/medical symptoms but also other important variables that must complete this complex picture (see Figure 1C).

Lately, the importance of a multidimensional approach has been emphasized to better comprehend frailty, not only as a physiopathological syndrome (Amanzio et al., 2017). According to this approach, the multidimensional prognostic index (MPI) could be considered a more comprehensive evaluation tool (Pilotto et al., 2013, 2020; Angleman et al., 2015), useful for the assessment of subjects with neurodegenerative disorders, from minor to major neurocognitive decline, with different frailty status (Amanzio et al., 2017).

### Frailty and Cognitive Functions: What Kind of Association?

Originally, the concept of frailty referred only to a physical condition; recently, it includes also a cognitive status, which could be related to a reduction of neurophysiological reserves. At present, cognition is considered a relevant domain for frailty comprehension and a novel target for the prevention of elderly dependency (Ruan et al., 2015). Indeed, cognitive frailty seems to be both an effect and a cause of physical frailty.

Physical frailty is considered a risk factor for Mild Cognitive Impairment (Boyle et al., 2010). In a 10-year longitudinal study, Raji et al. (2010) explored whether cognitive impairment could predict frailty risk in robust elderly. The authors suggested that robust older people with cognitive dysfunctions had a 9% higher chance to become frail per year, compared to the individuals with preserved cognition. 30.9% of the elderly with cognitive impairment fulfilled the criteria for weight loss from the first to the second follow-up, while the 25% fulfilled the criteria for slowness from the second to the last follow-up (Raji et al., 2010).

More recently, data from the Italian Longitudinal Study on Aging (ILSA) suggested that cognitive frailty increased risk of all common cause of mortality in older people, over a 3.5-year and 7-year follow-up (Solfrizzi et al., 2017). Cognitive impairment was found to be associated in a higher risk of adverse health outcomes also in The Singapore Longitudinal Aging Studies (SLAS), for which cognitive impairment resulted implicated in the increased prevalence and incidence of functional disability, poor quality of life, and mortality (Feng et al., 2017).

Cognitive impairment can be easily detected by administering neuropsychological cognitive tests, such as the MMSE. Exceeding the limit of the exclusive use of the MMSE, a small number of studies examined the association between specific cognitive functions and physical frailty (Canevelli et al., 2015), pointing out a relationship between a reduction in gait speed or grip strength and an impairment of attention and executive functions (Woollacott and Shumway-Cook, 2002; O’Halloran et al., 2014; Canevelli et al., 2015). These findings were supported by the results of a 9-year longitudinal study of 331 healthy women, which showed the association of executive functioning with frailty progression, suggesting that both impairments and declines in executive functioning were associated with risk of frailty onset (Gross et al., 2016). More recently, data from The Toledo Study for Healthy Aging (TSHA) demonstrated that deficit in executive functioning is a powerful predictor of frailty (increased risk of 13%), disability (increased risk of 11%), and mortality (increased risk of 7%) (Rosado-Artalejo et al., 2017).

Executive Functions (EFs) are a set of abilities that control thoughts and behaviors (Miyake and Friedman, 2012). They can be categorized into “cool” EFs, which involve conscious control of thoughts and actions in non-emotional conditions, and “hot” EFs, concerning goal-directed and future-oriented cognitive processes in contexts that elicit emotions, motivation, and tension (Poon, 2018). Although there is still no consensus regarding which are the cognitive functions that may or may not be included in the EFs (Poon, 2018), there is a general agreement that shifting, updating/monitoring, and inhibition are the core EFs (Diamond, 2013), which play a different and complementary role in performing complex executive tasks (Miyake et al., 2000). In order to comprehend the unity but also the heterogeneity of EFs, Miyake et al. (2000) proposed a structural model characterized by mental shifting, information monitoring and updating, and inhibition of preponderant responses. From these, higher-order EFs arise such as problem solving and planning (Lunt et al., 2012).

On the other hand, the term “executive dysfunction” refers to the inability to formulate, organize, and plan goal-directed behaviors and novel cognitive tasks (Parker et al., 2013).

Executive deficits are related to frontal network disruption and can occur in various diseases, including neurodegenerative disorders (Elliott, 2003). Several executive dysfunctions, evaluated by different methodologies and tools, have been reported in literature. The most common concern deficits in inhibitory control (inability to initiate an action or inhibit a predominant response and maintain attention), cognitive flexibility (shifting from a cognitive task to another), and monitoring (maintaining, organizing information and planning behavior) (Diamond, 2013).

Prefrontally mediated attentional-executive functions have been previously related to motor and other important features of physical frailty (Rosano et al., 2008; Amboni et al., 2013). Specific executive functions (EFs) associated with the medial prefrontal cortex - such as “action monitoring”—have been also considered in pre-frailty status in neurocognitive disorders due to Alzheimer’s disease (Amanzio et al., 2017).

### Frailty and Cognitive Impairment: The Need to Study the Case of Neurodegenerative Disorders

The first studies on frailty analyzed the association with cognitive impairment through the biomedical model (see Figures 1A,B). They emphasized how physical frailty, combined with cognitive impairment, is predictive of an increased risk of a poor prognosis. One of the first studies analyzed the association between physical frailty and a progressive cognitive decline (Samper-Ternent et al., 2008). In particular, 1370 subjects were studied and baseline values for physical frailty (according to Fried’s paradigm) and MMSE were observed after 3, 5, and 10 years. The results showed a substantial reduction of the mean of MMSE among frail individuals compared to pre-frail and robust ones.

Subsequent studies, while analyzing the presence of frailty with Fried’s paradigm, began to investigate different cognitive sub-domains, widening the focus of observation. This new approach, characterized by the assessment of the cognitive dimension of frailty, enabled to outline the neuropsychological profile of the elderly people analyzed. Some studies tried to investigate more deeply the relationship between cognitive domains and physical frailty (Canevelli et al., 2015). The authors pointed out that the best neuropsychological model to study the presence of frailty associated with cognitive impairment was the paradigms of attentional and executive functions (Canevelli et al., 2015). Interestingly, attention domain and executive functions seemed to be associated with frailty; on physical side, gait speed and grip strength were mainly related to cognitive impairment, with a particular role played by executive dysfunction (Lundin-Olsson et al., 1998; Patrick et al., 2002; Woollacott and Shumway-Cook, 2002; Harley et al., 2009; Kang et al., 2009; O’Halloran et al., 2011, 2014; Langlois et al., 2012; McGough et al., 2013; Shimada et al., 2013; Delrieu et al., 2016; Hooghiemstra et al., 2017; Sargent and Brown, 2017). In this direction, subjects in a pre-frail and frail status were less able to perform the “Sustained Attention to Response Task” (SART), compared to robust ones. Frailty patients made more commission errors and omissions, suggesting that their response monitoring ability could be impaired (Langner and Eickhoff, 2013; O’Halloran et al., 2014; Robertson et al., 2014).

Robertson et al. (2014), carried out a study on a community population of 50 years and older to analyze the association between frailty and different cognitive domains. The authors investigated the effect of each frailty indicator (according to the phenotypic model) on each cognitive domain analyzed (i.e., global cognition, memory, attention, executive functions, processing speed, and self-rated memory) through a multivariate linear regression. Results showed that asthenia was associated with global cognitive functioning, as was the decrease in handgrip strength. The latter was also associated with executive functioning, assessed by neuropsychological tests concerning reasoning, verbal fluency (phonemic) and response inhibition. Finally, walking speed was associated with different cognitive domains, such as attention, processing speed and executive functions.

Some other studies investigated the role played by mood changes on frailty (Mezuk et al., 2012; Espinoza et al., 2013; Paulson and Lichtenberg, 2013). Their results showed a possible association between depressive symptoms and frailty. In particular, depression could be both a cause and an effect of frailty (Robertson et al., 2013).

Even if these studies represent a first important attempt to describe the association between cognitive functions and physical frailty, there is still a need to assess frailty with a multidimensional approach (Pilotto and Ferrucci, 2011; Avelino-Silva et al., 2014; Sternberg and Bentur, 2014) (see Figure 1, C). Indeed, as underlined by the bio-psycho-social model, frailty is composed not only of physical aspects but also by cognitive and social components, which interact and influence each other (Mantovani et al., 2020).

Future studies should clarify the type of association between cognitive impairment and frailty, in order to implement effective treatments. It also remains to be determined whether this association is causal or shares aging-related mechanisms, such as neurodegeneration. To understand which one is predominant on the other, longitudinal studies should be set up in the field of primary prevention and in the continuum from MCI to major neurocognitive disorder. As well highlighted by Lyreskog (2018), a new framework that connects aging, cognitive decline, and neurodegenerative disease is needed. This new paradigm would be useful for “(a) adequately account for the effects that the processes of aging have on neural decline and disease, and (b) be helpful in identifying relevant groups of users and patients” (Lyreskog, 2018; page 57).

The progression of cognitive frailty towards neurodegenerative disorders is not currently clear. However, several longitudinal studies have investigated the possible association (Gómez-Gómez and Zapico, 2019). It has been suggested that classic aging mechanisms, such as oxidative stress, mitochondrial malfunction, and systemic inflammation could play a role in the pathogenesis of cognitive frailty and other associated neurodegenerative diseases (such as Alzheimer’s and Parkinson’ diseases) (Buchman et al., 2007; Ahmed et al., 2008; Robertson et al., 2013; Gómez-Gómez and Zapico, 2019). Frailty prevalence in neurodegenerative disorders was explored by The Comprehensive Assessment of Neurodegeneration and Dementia (COMPASS-ND) Study (Burt et al., 2019), which verified a prevalence rate equal to 11% and 14% according to the Frailty Index and the frailty phenotype, respectively. The prevalence of frailty in Alzheimer’s disease varied with a wide range from 11.1% to 50.0% (with a pooled prevalence of 31.9% in mild-moderate stages) (Kojima et al., 2017). A similar prevalence was found by Borda et al. (2019), who also observed a rate of 37.14% in a sample of patients with Lewy body dementia.

In Parkinson’s disease (PD) many motor and non-motor symptoms are difficult to explain in terms of a purely ascending degeneration process (Diederich and Parent, 2012), suggesting the need to consolidate the multidimensional elements of PD. In this perspective, the frailty model can be applied to motor disorders albeit with some caution. Frailty and PD may clinically overlap and screening PD patients for frailty may be warranted. Roland et al. (2012) found that correlation coefficients described relationships between PD-related characteristics and physical frailty according to the phenotype criteria. Indeed, frailty is common in PD (prevalence rate = 22.2%) and is associated with a more adverse clinical outcome (Peball et al., 2019). A review by Smith et al. (2019) also provided data in this direction: authors found a prevalence of frailty, which ranged from 29% to 67%.

All together, these findings suggest that the influence of underlying frailty should be considered when managing neurological conditions.

Therefore, a better understanding of cognitive factors, associated with multidisciplinary caring, will form the basis of assistance to frail elderly, with the following possible clinical relapses: slowing of functional decline, reduction of mortality/morbidity, improvement of the quality of life, and reduction of re-hospitalizations. Despite this, very few studies investigated the impact of cognitive functions (more specifically on executive functions) as a precipitating and perpetuating factor of frailty in subjects suffering from neurodegenerative disorders. The proposed mini-review focuses on common points characterizing executive dysfunction, neurocognitive and neurobiological factors potentially involved in frailty in such patients. In particular, the present study aims to investigate and address the following issues:

1. Since physical frailty and cognitive decline (in particular executive dysfunction) are two aspects strongly connected within neurodegenerative disorders (i.e., Alzheimer’s disease and Parkinson’s disease), are the latter duly taken into consideration in the literature?
2. Which of the frailty models are referred to in these studies (biomedical, bio-psycho-social)?
3. What kind of executive dysfunction are considered and with what neuropsychological tools are they detected?

### Selection of Studies

A systematic search strategy was implemented to identify studies on frailty, published until 31^*st*^ March 2020, across the online database most frequently used in the international literature (Medline database with PubMed literature search^1^). We used a single set of query terms: *Frailty* AND *Executive Functions* [ALL].

We adopted the “PRISMA Statement” in order to make the selection and data collection process clear (Liberati et al., 2009).

With this aim, we reviewed the relevant literature in order to ensure to select only papers regarding patients with mild or major neurocognitive disorders (DSM 5; American Psychiatric Association [APA], 2013) due to neurodegenerative disorders. We only selected original studies. Moreover, descriptive reviews, systematic reviews or meta-analyses were excluded.

**FIGURE 2:**
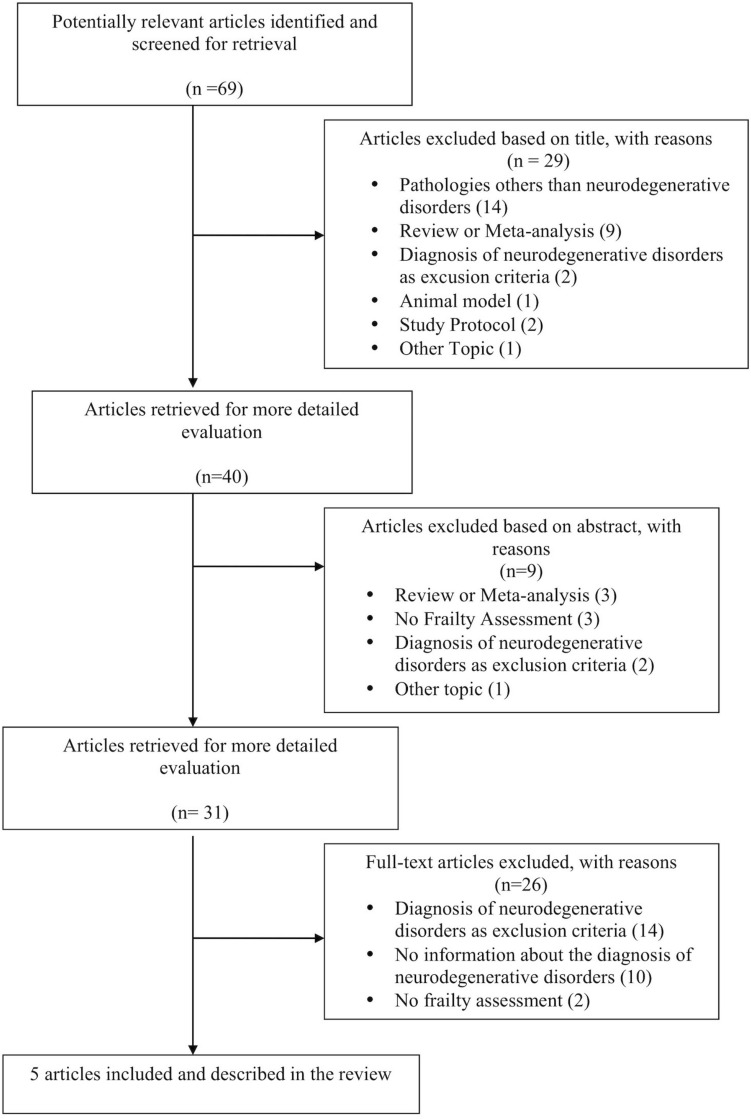
Article selection flow chart according to the PRISMA statement.

During the selection phase, we found 69 studies analyzing frailty in the above-mentioned pathologies. 64 studies were excluded because not consistent with the purpose of the review, while 5 were selected (see the flow chart in Figure 2 and the Supplementary Material for the selection of the articles and the reason for the exclusion).

### Description of the Selected Studies

The selected studies mainly concerned subjects with AD and PD, focusing in particular on the two most common neurodegenerative disorders (Xie et al., 2014). Four out of the five selected studies assessed frailty through the biomedical paradigm. In particular, three of those (Shimada et al., 2013; Chen et al., 2019; Lin et al., 2019) adopted Fried’s criteria, while one (Dutzi et al., 2017) used the model proposed by Rockwood et al. (2005) and Rockwood and Mitnitski (2007b).

Shimada et al. (2013) analyzed the relationship between physical frailty and Mild Cognitive Impairment (MCI) in 5104 community-dwelling persons aged 65 years and older (mean age 71 years).

The criteria used to define mild cognitive impairment are those reported by Petersen et al. (1999, 2001) for the “MCI-amnestic” type, which presents a high risk of conversion into a major neurocognitive disorder due to Alzheimer’s disease (Petersen et al., 1999; Grundman et al., 2004; Petersen and Negash, 2008);.

By adopting the phenotype model, Shimada et al. (2013) subdivided participants in respect of frailty status and level of cognitive impairment using the MMSE and 8 cognitive tests on memory, attention and executive functions, processing speed, and visuospatial skills. Particularly, the executive functioning – in terms of cognitive flexibility – was assessed through the trail making test (part A and B; Reitan and Wolfson, 1994).

The authors reported the presence of a frailty status in about 11% of the subjects and a MCI in about 19% of the participants. Considering the two aspects together, about 3% of subjects had both, frailty status and MCI, i.e., a cognitive frailty status (Kelaiditi et al., 2013).

Moreover, authors found that the subjects at higher risk for frailty were 80 years and older, with 9 years or less of education. As for cognitive impairment, the subjects with a higher probability of developing MCI were men, with 9 years or less of education. Finally, the co-occurrence of frailty and MCI (cognitive frailty) increased in relation with age and lower level of education.

The other two selected studies adopting the phenotypic model analyzed the relationship between physical frailty and cognitive impairment in patients with PD (Chen et al., 2019; Lin et al., 2019).

Chen et al. (2019) investigated structural brain changes in relation to physical frailty and cognitive decline in sixty-one PD patients (mean age 62.61 ± 8.59 years), by using MRI. Voxel-wise multiple linear regression analyses were carried out in order to identify the overlapping areas of gray matter volume decrease concerning such aspects.

Frailty was assessed by adopting Fried’s criteria. Several cognitive domains, such as attention, memory, language, visuospatial skills, and executive functions, were neuropsychologically evaluated. In particular, EFs were investigated, as indicated by the authors, by using some Wechsler Adult Intelligence Scale-III subtests (picture arrangement, arithmetic, digit symbol coding, and matrix reasoning) (Wechsler et al., 2002), and by the abstract thinking scores from the Cognitive Ability Screening Instrument (Lin et al., 2012).

The authors identified the lateral occipital cortex as an overlapping region of physical frailty and cognitive impairment. Specifically, an overlapping region was observed in the left lateral occipital cortex for every cognitive domain in relation to frailty. This cerebral region is part of the ventral object-based visual pathway (Mishkin et al., 1983), whose decrease in thickness had previously been identified in PD patients in relation to impaired cognitive functioning, in particular visuospatial skills, memory, and executive functions (Pereira et al., 2014).

Moreover, an additional overlapping region relating to the superior frontal gyrus had been identified in connection with executive functioning and frailty. These findings highlighted how frailty and cognitive decline are connected in the brain (Chen et al., 2019).

As a precaution, considering the elements of difficult disambiguation between frailty and PD, it is appropriate to consider the correlations between frailty and cognitive impairments observed in the study by Chen and collaborators related to the pathophysiology (e.g., alpha synuclein in the brain) rather than a sign of frailty.

Finally, by adopting Fried’s criteria, Lin et al. (2019) divided their sample of 76 PD patients (mean age 62.64 ± 9.23 years) into two groups: “with physical frailty” (38.2%) and “without physical frailty” (61.8%). PD patients with frailty were significantly older, showed worse disease severity, and poorer cognitive functions compared to robust ones. The neuropsychological assessment was the same carried out in Chen et al.’s study (2019).

A stepwise logistic regression analysis indicated how impaired executive functions increased considerably the risk of physical frailty.

In light of these results, the authors suggested that assessing cognitive functions in PD patients might be a useful approach to identify the subjects at greatest risk of developing frailty and to prevent negative outcomes through targeted strategies of intervention (Lin et al., 2019).

Dutzi et al. (2017) assessed frailty by using the model proposed by Rockwood et al. (2005). The authors investigated cognitive changes following hospital rehabilitation in 154 patients (mean age 83.7 ± 5.9) with mild and major neurocognitive disorder, with different etiopathogenesis [Alzheimer’s disease (AD) prevalently]. They considered several aspects that could affect rehabilitation, including cognitive functioning, independence in basic activities of daily living (bADL), and frailty status. Particularly, frailty was evaluated using the Clinical Frailty Scale (CFS) (Rockwood et al., 2005), which allows the clinician to assess the patient’s degree of frailty through clinical data. This tool correlates strongly with FI but is faster and easier to administer (Rockwood et al., 2005). The executive functioning was evaluated by the verbal fluency and the modified version of the trail making test, from Nuremberg Gerontopsychological Inventory (Oswald and Fleischmann, 1985). The verbal fluency test is considered a task for the assessment of cognitive flexibility (Diamond, 2013), as well as the trail making test (Lezak et al., 2004).

The authors found that patients presenting a worse frailty status and lower functional independence during the admission were those who did not benefit from cognitive rehabilitation.

They suggested that frailty and deficit in the bADL may have played an important role in the worsening of cognitive decline and in the ineffectiveness of the rehabilitation intervention (Dutzi et al., 2017).

As previously mentioned, 4 out of the 5 selected studies analyzed frailty by adopting the biomedical models. Only one study (Amanzio et al., 2017) provided for the assessment of frailty through the bio-psycho-social model, highlighting its multidimensionality (Pilotto et al., 2020). Amanzio et al. (2017) investigated the association among a multidimensional assessment of frailty, executive dysfunction, and specific cognitive and behavioral changes, using an overall neuropsychological battery in sixty patients with mild and major neurocognitive disorders due to AD (mean age 66.62 ± 6.80).

The authors used the MPI for a comprehensive assessment of frailty (EIP-AHA–European Innovation Partnership on Active and Healthy Ageing, 2013; Pilotto et al., 2013, 2020; Angleman et al., 2015). This tool not only takes into consideration the clinical, functional, neuropsychological, and nutritional status, but also gives information on the associated pathologies and pharmacological therapies, and on the social support network (Pilotto and Ferrucci, 2011, Pilotto et al., 2013, 2020). Executive functions, in terms of self-monitoring, were assessed through the metacognitive version of the Wisconsin Card Sorting test (m-WCST: Koren et al., 2006). This version differs from the original one as the subject is asked to answer two questions: to assess his or her online self-monitoring (“What is your degree of confidence in this answer?”) and to control abilities (“Do you want to take this response into account in your total score?”) (see Amanzio et al., 2017).

These findings suggested that also a pre-frailty status was associated with metacognitive-executive dysfunction, in terms of action monitoring in MCI-likely due to AD and AD patients. Specifically, it was observed a significant association between the MPI and monitoring resolution at the m-WCST, where patients failed to distinguish between correct and incorrect sorts. These results were specific and not influenced by other cognitive functions such as global cognition, memory, language comprehension, and non-verbal reasoning, with the exception of the selective attention task that reached a near significance level. Moreover, taking into account the MPI scores, this study demonstrated an involvement of mood depression changes, apathy, disinhibition, and a reduced awareness of IADL, associated with a higher frailty status (Amanzio et al., 2017).

Since apathy, disinhibition, and executive dysfunction seemed to be attributable to the malfunction of the same brain network (Masterman and Cummings, 1997; Bonelli and Cummings, 2007), the authors hypothesized that pre-frailty might also be due to a dysfunction of the medial prefrontal-ventral striatal network (Amanzio et al., 2017).

## Conclusion

The studies analyzed in this mini-review highlighted how physical frailty and cognitive decline, particularly executive dysfunction, are two aspects heavily connected within neurodegenerative disorders (i.e., AD and PD).

Several cognitive domains have been taken into account in the selected studies due to the lack of a univocal definition of EFs, assessed by different neuropsychological instruments.

The analyzed studies showed that frailty is related to executive dysfunction, in terms of cognitive flexibility (Shimada et al., 2013; Dutzi et al., 2017) and self-monitoring (Amanzio et al., 2017) in neurocognitive disorders.

In our opinion, the Wechsler Adult Intelligence Scale-III (WAIS-III) subtests, used by Chen et al. (2019) and Lin et al. (2019), are not the gold-standard instruments to assess EFs, as WAIS-III was created for the evaluation of reasoning and intellectual abilities (Wechsler, 1997).

However, as reported by Robertson et al. (2014), several cognitive functions such as global cognition, attention, executive functions—including reasoning—and memory are associated with frailty status. These results confirm the hypothesis that there is a relation between frailty and cognitive decline in different domains, even within neurodegenerative disorders (such as PD).

Previous researches had shown a strong association between physical frailty and the incident of neurocognitive disorders, such as AD, MCI (Panza et al., 2015; Xu et al., 2015; Kojima et al., 2016), and cerebral vascular diseases (Avila-Funes et al., 2012). Frailty and cognitive impairment share several risk factors such as age-related chronic diseases, inflammation or cardiovascular problems (Robertson et al., 2013).

In a recent work of systematic review and meta-analysis, Borges et al. (2019) investigated the relationship between physical frailty and cognitive impairment, highlighting how frailty seemed to be one of the greatest risk factors for the development of major neurocognitive disorders.

However, it is important to underline how, to date, the studies have not clarified the direction of the association between frailty and the presence of a cognitive impairment yet. In particular, it is the presence of frailty that determines cognitive impairment or vice versa?

In our opinion, given the multidimensional nature of frailty, the bio-psycho-social model is the most appropriate paradigm for the evaluation and management of frail older people with cognitive decline.

Longitudinal studies may be the most correct approach to assess the presence of cognitive disorders many years before the development of frailty itself. Further studies will be important to better characterized this association over time and replicate these findings in a larger group of patients. Analyzing the association between frailty and cognitive dysfunction in this at-risk population, would allow to develop specific physical and/or cognitive empowerment and rehabilitation measures.

## Author Contributions

MB wrote the manuscript, developed the search strategy, revised the images and figures, and took part in the review and critique processes. SP wrote the manuscript and took part in the review and critique processes. GEC wrote and edited the manuscript, revised the images and figures. MA conceived the content of the review, developed the search strategy, wrote the first draft of the manuscript, proceeded to extend the theoretical models of frailty associated with executive functions, supervised subsequent changes and took part in the critique processes. All authors contributed to the article and approved the submitted version.

## Conflict of Interest

The authors declare that the research was conducted in the absence of any commercial or financial relationships that could be construed as a potential conflict of interest.

## Footnotes

1 http://www.ncbi.nlm.nih.gov/pubmed
