# Supplementary material for: A Possible Association Between Executive Dysfunction and Frailty in Patients With Neurocognitive Disorders
Source: Front Psychol. 2020 Nov 11;11:554307. doi: 10.3389/fpsyg.2020.554307 (PMC7685991; doi:10.3389/fpsyg.2020.554307)
Supplement: Supplementary file 1 [file Data_Sheet_1.ZIP › Bartoli et al. Mini-review_Supplementary Material_PRISMA Selection/Bartoli et al. Mini-review_3_Supplemental Material_Full-text selection.docx]

**Supplemental Material:**

Full Text Selection according to the PRISMA Statement

Studies found by searching keywords. Included studies (full text selection) are shown in bold characters. For the excluded studies the reason for exclusion is reported in square parentheses.

Key search with no time limit: “Frailty” AND “Executive Functions”

## Search results

### Included: 5/31

3. [Excluded: Diagnosis of neurodegenerative disorders as exclusion criteria]

[Motoric cognitive risk syndrome is associated with processing speed and executive function, but not delayed free recall memory: The Korean frailty and aging cohort study (KFACS).](https://www.ncbi.nlm.nih.gov/pubmed/31786409)

Shim H, Kim M, Won CW.

Arch Gerontol Geriatr. 2019 Nov 19;87:103990. doi: 10.1016/j.archger.2019.103990. [Epub ahead of print]

**5.**

[Associations Between Cognitive Functions and Physical Frailty in Patients With Parkinson's Disease.](https://www.ncbi.nlm.nih.gov/pubmed/31736737)

**Lin WC, Huang YC, Leong CP, Chen MH, Chen HL, Tsai NW, Tso HH, Chen PC, Lu CH.**

**Front Aging Neurosci. 2019 Oct 30;11:283. doi: 10.3389/fnagi.2019.00283. eCollection 2019.**

6. [Excluded: No information about the Diagnosis of Neurodegenerative Disorders]

[Effects of a Multicomponent Frailty Prevention Program in Prefrail Community-Dwelling Older Persons: A Randomized Controlled Trial.](https://www.ncbi.nlm.nih.gov/pubmed/31706917)

Yu R, Tong C, Ho F, Woo J.

J Am Med Dir Assoc. 2020 Feb;21(2):294.e1-294.e10. doi: 10.1016/j.jamda.2019.08.024. Epub 2019 Nov 7.

7. [Excluded: Diagnosis of neurodegenerative disorder as exclusion criteria]

[Exposure to Ambient Air Pollution and Cognitive Impairment in Community-Dwelling Older Adults: The Korean Frailty and Aging Cohort Study.](https://www.ncbi.nlm.nih.gov/pubmed/31591354)

Shin J, Han SH, Choi J.

Int J Environ Res Public Health. 2019 Oct 7;16(19). pii: E3767. doi: 10.3390/ijerph16193767.

# 9. [Excluded: No information about the Diagnosis of Neurodegenerative Disease]

[Identification of five frailty profiles in community-dwelling individuals aged 50-75: A latent class analysis of the SUCCEED survey data.](https://www.ncbi.nlm.nih.gov/pubmed/31351514)

Segaux L, Oubaya N, Broussier A, Baude M, Canouï-Poitrine F, Naga H, Laurent M, Leissing-Desprez C, Audureau E, Ferrat E, Chailloleau C, Fromentin I, David JP, Bastuji-Garin S.

Maturitas. 2019 Sep;127:1-11. doi: 10.1016/j.maturitas.2019.05.007. Epub 2019 May 20.

10. [Excluded: Diagnosis of neurodegenerative disorders as exclusion criteria]

[Assessing the impact of physical exercise on cognitive function in older medical patients during acute hospitalization: Secondary analysis of a randomized trial.](https://www.ncbi.nlm.nih.gov/pubmed/31276501)

Sáez de Asteasu ML, Martínez-Velilla N, Zambom-Ferraresi F, Casas-Herrero Á, Cadore EL, Galbete A, Izquierdo M.

PLoS Med. 2019 Jul 5;16(7):e1002852. doi: 10.1371/journal.pmed.1002852. eCollection 2019 Jul.

13. [Excluded: No frailty assessment]

[Polypharmacy and gait speed in individuals with mild cognitive impairment.](https://www.ncbi.nlm.nih.gov/pubmed/31106973)

Umegaki H, Yanagawa M, Komiya H, Matsubara M, Fujisawa C, Suzuki Y, Kuzuya M.

Geriatr Gerontol Int. 2019 Aug;19(8):730-735. doi: 10.1111/ggi.13688. Epub 2019 May 20.

**19.**

[Reduced lateral occipital gray matter volume is associated with physical frailty and cognitive impairment in Parkinson's disease.](https://www.ncbi.nlm.nih.gov/pubmed/30523452)

**Chen YS, Chen HL, Lu CH, Chen MH, Chou KH, Tsai NW, Yu CC, Chiang PL, Lin WC.**

**Eur Radiol. 2019 May;29(5):2659-2668. doi: 10.1007/s00330-018-5855-7. Epub 2018 Dec 6.**

20. [Excluded: Diagnosis of neurodegenerative disorders as exclusion criteria]

[Cognitive Frailty is Associated with Fall-Related Fracture among Older People.](https://www.ncbi.nlm.nih.gov/pubmed/30498829)

Tsutsumimoto K, Doi T, Makizako H, Hotta R, Nakakubo S, Makino K, Suzuki T, Shimada H.

J Nutr Health Aging. 2018;22(10):1216-1220. doi: 10.1007/s12603-018-1131-4.

23. [Excluded: Diagnosis of neurodegenerative disorders as exclusion criteria]]

[Effects of Resistance Exercise Training on Cognitive **Function** and Physical Performance in Cognitive **Frailty**: A Randomized Controlled Trial.](https://www.ncbi.nlm.nih.gov/pubmed/30272098)

Yoon DH, Lee JY, Song W.

J Nutr Health Aging. 2018;22(8):944-951. doi: 10.1007/s12603-018-1090-9.

24. [Excluded: Diagnosis of neurodegenerative disorders as exclusion criteria]]

[Cognitive **Frailty** Predicts Incident Dementia among Community-Dwelling Older People.](https://www.ncbi.nlm.nih.gov/pubmed/30200236)

Shimada H, Doi T, Lee S, Makizako H, Chen LK, Arai H.

J Clin Med. 2018 Aug 30;7(9). pii: E250. doi: 10.3390/jcm7090250.

26. [Excluded: Diagnosis of neurodegenerative disorders as exclusion criteria]

[Comparison of cognitive **functions** among frail and prefrail older adults: a clinical perspective.](https://www.ncbi.nlm.nih.gov/pubmed/29954464)

da Silva Alves BB, de Oliveira Barbosa E, de Moraes Pimentel D, Carneiro LSF, Rodrigues ACMA, Deslandes AC, Alves MR, Rodrigues VD, Pereira EL, de Paula AMB, Pupe CCB, Monteiro-Junior RS.

Int Psychogeriatr. 2019 Feb;31(2):297-301. doi: 10.1017/S1041610218000765. Epub 2018 Jun 29.

30. [Excluded: Diagnosis of neurodegenerative disorders as exclusion criteria]]

[Cognitive **Frailty** and Incidence of Dementia in Older Persons.](https://www.ncbi.nlm.nih.gov/pubmed/29405232)

Shimada H, Makizako H, Tsutsumimoto K, Doi T, Lee S, Suzuki T.

J Prev Alzheimers Dis. 2018;5(1):42-48. doi: 10.14283/jpad.2017.29.

**33.**

[**Neuropsychological Correlates of Pre-Frailty in Neurocognitive Disorders: A Possible Role for Metacognitive Dysfunction and Mood Changes.**](https://www.ncbi.nlm.nih.gov/pubmed/29188218)

**Amanzio M, Palermo S, Zucca M, Rosato R, Rubino E, Leotta D, Bartoli M, Rainero I.**

**Front Med (Lausanne). 2017 Nov 15;4:199. doi: 10.3389/fmed.2017.00199. eCollection 2017.**

34. [Excluded: No information about the Diagnosis of Neurodegenerative Disease]

[Global Performance of **Executive Function** Is Predictor of Risk of **Frailty** and Disability in Older Adults.](https://www.ncbi.nlm.nih.gov/pubmed/29083438)

Rosado-Artalejo C, Carnicero JA, Losa-Reyna J, Castillo C, Cobos-Antoranz B, Alfaro-Acha A, Rodríguez-Mañas L, García-García FJ.

J Nutr Health Aging. 2017;21(9):980-987. doi: 10.1007/s12603-017-0895-2.

**35.**

[**Cognitive Change in Rehabilitation Patients with Dementia: Prevalence and Association with Rehabilitation Success.**](https://www.ncbi.nlm.nih.gov/pubmed/28984597)

**Dutzi I, Schwenk M, Kirchner M, Bauer JM, Hauer K.**

**J Alzheimers Dis. 2017;60(3):1171-1182. doi: 10.3233/JAD-170401.**

41. [Excluded: Diagnosis of neurodegenerative disorders as exclusion criteria]]

[Gait Speed and Grip Strength Reflect Cognitive Impairment and Are Modestly Related to Incident Cognitive Decline in Memory Clinic Patients With Subjective Cognitive Decline and Mild Cognitive Impairment: Findings From the 4C Study.](https://www.ncbi.nlm.nih.gov/pubmed/28177065)

Hooghiemstra AM, Ramakers IHGB, Sistermans N, Pijnenburg YAL, Aalten P, Hamel REG, Melis RJF, Verhey FRJ, Olde Rikkert MGM, Scheltens P, van der Flier WM; 4C Study Group.

J Gerontol A Biol Sci Med Sci. 2017 Jun 1;72(6):846-854. doi: 10.1093/gerona/glx003.

46. [Excluded: No frailty assessment]

[The Early Indicators of Functional Decrease in Mild Cognitive Impairment.](https://www.ncbi.nlm.nih.gov/pubmed/27570509)

Kubicki A, Fautrelle L, Bourrelier J, Rouaud O, Mourey F.

Front Aging Neurosci. 2016 Aug 12;8:193. doi: 10.3389/fnagi.2016.00193. eCollection 2016.

47. [Excluded: Diagnosis of neurodegenerative disorders as exclusion criteria]]

[Impact of Cognitive **Frailty** on Daily Activities in Older Persons.](https://www.ncbi.nlm.nih.gov/pubmed/27499306)

Shimada H, Makizako H, Lee S, Doi T, Lee S, Tsutsumimoto K, Harada K, Hotta R, Bae S, Nakakubo S, Harada K, Suzuki T.

J Nutr Health Aging. 2016;20(7):729-35. doi: 10.1007/s12603-016-0685-2.

48. [Excluded: Diagnosis of neurodegenerative disorders as exclusion criteria]

[Declines and Impairment in **Executive Function** Predict Onset of Physical **Frailty**.](https://www.ncbi.nlm.nih.gov/pubmed/27084314)

Gross AL, Xue QL, Bandeen-Roche K, Fried LP, Varadhan R, McAdams-DeMarco MA, Walston J, Carlson MC.

J Gerontol A Biol Sci Med Sci. 2016 Dec;71(12):1624-1630. Epub 2016 Apr 15.

49. [Excluded: Diagnosis of neurodegenerative disorders as exclusion criteria]]

[Cognitive impairment is associated with the absence of fear of falling in community-dwelling frail older adults.](https://www.ncbi.nlm.nih.gov/pubmed/26792588)

Shirooka H, Nishiguchi S, Fukutani N, Tashiro Y, Nozaki Y, Hirata H, Yamaguchi M, Tasaka S, Matsushita T, Matsubara K, Aoyama T.

Geriatr Gerontol Int. 2017 Feb;17(2):232-238. doi: 10.1111/ggi.12702. Epub 2016 Jan 21.

51. [Excluded: No information about the Diagnosis of Neurodegenerative Disease]

[Stress Regulation as a Link between **Executive Function** and Pre-**Frailty** in Older Adults.](https://www.ncbi.nlm.nih.gov/pubmed/26412287)

Roiland RA, Lin F, Phelan C, Chapman BP.

J Nutr Health Aging. 2015 Oct;19(8):828-38. doi: 10.1007/s12603-015-0476-1.

55. [Excluded: No information about the Diagnosis of Neurodegenerative Disease]

[Reconceptualizing balance: attributes associated with balance performance.](https://www.ncbi.nlm.nih.gov/pubmed/24952097)

Thomas JC, Odonkor C, Griffith L, Holt N, Percac-Lima S, Leveille S, Ni P, Latham NK, Jette AM, Bean JF.

Exp Gerontol. 2014 Sep;57:218-23. doi: 10.1016/j.exger.2014.06.012. Epub 2014 Jun 18.

56. [Excluded: No information about the Diagnosis of Neurodegenerative Disease]

[Linking cognition and **frailty** in middle and old age: metabolic syndrome matters.](https://www.ncbi.nlm.nih.gov/pubmed/24733716)

Lin F, Roiland R, Chen DG, Qiu C.

Int J Geriatr Psychiatry. 2015 Jan;30(1):64-71. doi: 10.1002/gps.4115. Epub 2014 Apr 15.

58. [Excluded: No information about the Diagnosis of Neurodegenerative Disease]

[Serum 25-hydroxyvitamin D is associated with cognitive **executive function** in Dutch prefrail and frail elderly: a cross-sectional study exploring the associations of 25-hydroxyvitamin D with glucose metabolism, cognitive performance and depression.](https://www.ncbi.nlm.nih.gov/pubmed/23921196)

Brouwer-Brolsma EM, van de Rest O, Tieland M, van der Zwaluw NL, Steegenga WT, Adam JJ, van Loon LJ, Feskens EJ, de Groot LC.

J Am Med Dir Assoc. 2013 Nov;14(11):852.e9-17. doi: 10.1016/j.jamda.2013.06.010. Epub 2013 Aug 3.

**60.**

[**Combined prevalence of frailty and mild cognitive impairment in a population of elderly Japanese people.**](https://www.ncbi.nlm.nih.gov/pubmed/23669054)

**Shimada H, Makizako H, Doi T, Yoshida D, Tsutsumimoto K, Anan Y, Uemura K, Ito T, Lee S, Park H, Suzuki T.**

**J Am Med Dir Assoc. 2013 Jul;14(7):518-24. doi: 10.1016/j.jamda.2013.03.010. Epub 2013 May 10.**

62. [Excluded: No information about the Diagnosis of Neurodegenerative Disease]

[Sustained attention and **frailty** in the older adult population.](https://www.ncbi.nlm.nih.gov/pubmed/23525545)

O'Halloran AM, Finucane C, Savva GM, Robertson IH, Kenny RA.

J Gerontol B Psychol Sci Soc Sci. 2014 Mar;69(2):147-56. doi: 10.1093/geronb/gbt009. Epub 2013 Mar 22.

63. [Excluded: Diagnosis of neurodegenerative disorders as exclusion criteria]

[Benefits of physical exercise training on cognition and quality of life in frail older adults.](https://www.ncbi.nlm.nih.gov/pubmed/22929394)

Langlois F, Vu TT, Chassé K, Dupuis G, Kergoat MJ, Bherer L.

J Gerontol B Psychol Sci Soc Sci. 2013 May;68(3):400-4. doi: 10.1093/geronb/gbs069. Epub 2012 Aug 28.

64. [Excluded: No information about the Diagnosis of Neurodegenerative Disease]

[The multiple dimensions of **frailty**: physical capacity, cognition, and quality of life.](https://www.ncbi.nlm.nih.gov/pubmed/22717010)

Langlois F, Vu TT, Kergoat MJ, Chassé K, Dupuis G, Bherer L.

Int Psychogeriatr. 2012 Sep;24(9):1429-36. doi: 10.1017/S1041610212000634. Epub 2012 Apr 25.

66. [Excluded: Diagnosis of neurodegenerative disorders as exclusion criteria]]

[Geriatric syndromes in older homeless adults.](https://www.ncbi.nlm.nih.gov/pubmed/21879368)

Brown RT, Kiely DK, Bharel M, Mitchell SL.

J Gen Intern Med. 2012 Jan;27(1):16-22. doi: 10.1007/s11606-011-1848-9. Epub 2011 Aug 31.

68. [Excluded: No information about the Diagnosis of Neurodegenerative Disease]

[Validation and comparison of two **frailty** indexes: The MOBILIZE Boston Study.](https://www.ncbi.nlm.nih.gov/pubmed/19682112)

Kiely DK, Cupples LA, Lipsitz LA.

J Am Geriatr Soc. 2009 Sep;57(9):1532-9. doi: 10.1111/j.1532-5415.2009.02394.x. Epub 2009 Jul
