# Supplementary material for: A Possible Association Between Executive Dysfunction and Frailty in Patients With Neurocognitive Disorders
Source: Front Psychol. 2020 Nov 11;11:554307. doi: 10.3389/fpsyg.2020.554307 (PMC7685991; doi:10.3389/fpsyg.2020.554307)
Supplement: Supplementary file 1 [file Data_Sheet_1.ZIP › Bartoli et al. Mini-review_Supplementary Material_PRISMA Selection/Bartoli et al. Mini-review_1_Supplemental Material_Title Selection.docx]

**Supplemental Material:**

Title Selection according to the PRISMA Statement

Studies found by searching keywords. Included studies (title selection) are shown in bold characters. For the excluded studies the reason for exclusion is reported in square parentheses.

Key search with no time limit: “Frailty” AND “Executive Functions”

## Search results

### Included: 40/69

1. [Excluded: Study Protocol]

[Motor, cognitive and mobility deficits in 1000 geriatric patients: protocol of a quantitative observational study before and after routine clinical geriatric treatment - the ComOn-study.](https://www.ncbi.nlm.nih.gov/pubmed/32028945)

Geritz J, Maetzold S, Steffen M, Pilotto A, Corrà MF, Moscovich M, Rizzetti MC, Borroni B, Padovani A, Alpes A, Bang C, Barcellos I, Baron R, Bartsch T, Becktepe JS, Berg D, Bergeest LM, Bergmann P, Bouça-Machado R, Drey M, Elshehabi M, Farahmandi S, Ferreira JJ, Franke A, Friederich A, Geisler C, Hüllemann P, Gierthmühlen J, Granert O, Heinzel S, Heller MK, Hobert MA, Hofmann M, Jemlich B, Kerkmann L, Knüpfer S, Krause K, Kress M, Krupp S, Kudelka J, Kuhlenbäumer G, Kurth R, Leypoldt F, Maetzler C, Maia LF, Moewius A, Neumann P, Niemann K, Ortlieb CT, Paschen S, Pham MH, Puehler T, Radloff F, Riedel C, Rogalski M, Sablowsky S, Schanz EM, Schebesta L, Schicketmüller A, Studt S, Thieves M, Tönges L, Ullrich S, Urban PP, Vila-Chã N, Wiegard A, Warmerdam E, Warnecke T, Weiss M, Welzel J, Hansen C, Maetzler W.

BMC Geriatr. 2020 Feb 6;20(1):45. doi: 10.1186/s12877-020-1445-z.

2. [Excluded: Pathologies others than neurodegenerative disorders]

[Neurocognitive and Muscular Capacities Are Associated with Frailty in Adults with Cirrhosis.](https://www.ncbi.nlm.nih.gov/pubmed/31982996)

Murphy SL, Richardson JK, Blackwood J, Martinez B, Tapper EB.

Dig Dis Sci. 2020 Jan 25. doi: 10.1007/s10620-020-06099-4. [Epub ahead of print]

**3.**

[Motoric cognitive risk syndrome is associated with processing speed and executive function, but not delayed free recall memory: The Korean frailty and aging cohort study (KFACS).](https://www.ncbi.nlm.nih.gov/pubmed/31786409)

**Shim H, Kim M, Won CW.**

**Arch Gerontol Geriatr. 2019 Nov 19;87:103990. doi: 10.1016/j.archger.2019.103990. [Epub ahead of print]**

**4.**

[Neuropsychology of aging.](https://www.ncbi.nlm.nih.gov/pubmed/31753131)

**Cohen RA, Marsiske MM, Smith GE.**

**Handb Clin Neurol. 2019;167:149-180. doi: 10.1016/B978-0-12-804766-8.00010-8.**

**5.**

[Associations Between Cognitive Functions and Physical Frailty in Patients With Parkinson's Disease.](https://www.ncbi.nlm.nih.gov/pubmed/31736737)

**Lin WC, Huang YC, Leong CP, Chen MH, Chen HL, Tsai NW, Tso HH, Chen PC, Lu CH.**

**Front Aging Neurosci. 2019 Oct 30;11:283. doi: 10.3389/fnagi.2019.00283. eCollection 2019.**

**6.**

[Effects of a Multicomponent Frailty Prevention Program in Prefrail Community-Dwelling Older Persons: A Randomized Controlled Trial.](https://www.ncbi.nlm.nih.gov/pubmed/31706917)

**Yu R, Tong C, Ho F, Woo J.**

**J Am Med Dir Assoc. 2020 Feb;21(2):294.e1-294.e10. doi: 10.1016/j.jamda.2019.08.024. Epub 2019 Nov 7.**

**7.**

[Exposure to Ambient Air Pollution and Cognitive Impairment in Community-Dwelling Older Adults: The Korean Frailty and Aging Cohort Study.](https://www.ncbi.nlm.nih.gov/pubmed/31591354)

**Shin J, Han SH, Choi J.**

**Int J Environ Res Public Health. 2019 Oct 7;16(19). pii: E3767. doi: 10.3390/ijerph16193767.**

8. [Excluded: Pathologies others than neurodegenerative disorders]

[Neuro-Psychiatric Alterations in Patients with Diabetic Foot Syndrome.](https://www.ncbi.nlm.nih.gov/pubmed/31577209)

Navarro-Flores E, Pérez-Ros P, Martínez-Arnau FM, Julían-Rochina I, Cauli O.

CNS Neurol Disord Drug Targets. 2019;18(8):598-608. doi: 10.2174/1871527318666191002094406.

# 9.

[Identification of five frailty profiles in community-dwelling individuals aged 50-75: A latent class analysis of the SUCCEED survey data.](https://www.ncbi.nlm.nih.gov/pubmed/31351514)

**Segaux L, Oubaya N, Broussier A, Baude M, Canouï-Poitrine F, Naga H, Laurent M, Leissing-Desprez C, Audureau E, Ferrat E, Chailloleau C, Fromentin I, David JP, Bastuji-Garin S.**

**Maturitas. 2019 Sep;127:1-11. doi: 10.1016/j.maturitas.2019.05.007. Epub 2019 May 20.**

**10.**

[Assessing the impact of physical exercise on cognitive function in older medical patients during acute hospitalization: Secondary analysis of a randomized trial.](https://www.ncbi.nlm.nih.gov/pubmed/31276501)

**Sáez de Asteasu ML, Martínez-Velilla N, Zambom-Ferraresi F, Casas-Herrero Á, Cadore EL, Galbete A, Izquierdo M.**

**PLoS Med. 2019 Jul 5;16(7):e1002852. doi: 10.1371/journal.pmed.1002852. eCollection 2019 Jul.**

# 11. [Excluded: Review]

[Physical activity programs for older people in the community receiving home care services: systematic review and meta-analysis.](https://www.ncbi.nlm.nih.gov/pubmed/31239654)

Burton E, Farrier K, Galvin R, Johnson S, Horgan NF, Warters A, Hill KD.

Clin Interv Aging. 2019 Jun 6;14:1045-1064. doi: 10.2147/CIA.S205019. eCollection 2019.

12. [Excluded: Diagnosis of neurodegenerative disorders as exclusion criteria]

[Frailty effects on non-demented cognitive trajectories are moderated by sex and Alzheimer's genetic risk.](https://www.ncbi.nlm.nih.gov/pubmed/31221191)

Thibeau S, McDermott K, McFall GP, Rockwood K, Dixon RA.

Alzheimers Res Ther. 2019 Jun 21;11(1):55. doi: 10.1186/s13195-019-0509-9.

**13.**

[Polypharmacy and gait speed in individuals with mild cognitive impairment.](https://www.ncbi.nlm.nih.gov/pubmed/31106973)

**Umegaki H, Yanagawa M, Komiya H, Matsubara M, Fujisawa C, Suzuki Y, Kuzuya M.**

**Geriatr Gerontol Int. 2019 Aug;19(8):730-735. doi: 10.1111/ggi.13688. Epub 2019 May 20.**

14. [Excluded: Review]

[Diabetes-Related Dementia.](https://www.ncbi.nlm.nih.gov/pubmed/31062329)

Hanyu H.

Adv Exp Med Biol. 2019;1128:147-160. doi: 10.1007/978-981-13-3540-2_8. Review.

**15.**

[Sarcopenia Is Associated with Cognitive Impairment Mainly Due to Slow Gait Speed: Results from the Korean Frailty and Aging Cohort Study (KFACS).](https://www.ncbi.nlm.nih.gov/pubmed/31035553)

**Kim M, Won CW.**

**Int J Environ Res Public Health. 2019 Apr 27;16(9). pii: E1491. doi: 10.3390/ijerph16091491.**

16. [Excluded: Diagnosis of neurodegenerative disorders as exclusion criteria]

[Sleep quality mediates the relationship between frailty and cognitive dysfunction in non-demented middle aged to older adults.](https://www.ncbi.nlm.nih.gov/pubmed/31006402)

Kaur S, Banerjee N, Miranda M, Slugh M, Sun-Suslow N, McInerney KF, Sun X, Ramos AR, Rundek T, Sacco RL, Levin BE.

Int Psychogeriatr. 2019 Jun;31(6):779-788. doi: 10.1017/S1041610219000292. Epub 2019 Apr 22.

**17.**

[The relationship between basic, instrumental, and advanced activities of daily living and executive functioning in geriatric patients with neurocognitive disorders.](https://www.ncbi.nlm.nih.gov/pubmed/30761619)

**Cornelis E, Gorus E, Van Schelvergem N, De Vriendt P.**

**Int J Geriatr Psychiatry. 2019 Jun;34(6):889-899. doi: 10.1002/gps.5087. Epub 2019 Mar 27.**

18. [Excluded: Pathologies others than neurodegenerative disorders]

[Targeting HIV-Related Neurocognitive Impairments with Cognitive Training Strategies: Insights from the Cognitive Aging Literature.](https://www.ncbi.nlm.nih.gov/pubmed/30710223)

Vance DE, Fazeli PL, Cheatwood J, Nicholson C, Morrison S, Moneyham LD.

Curr Top Behav Neurosci. 2019 Feb 2. doi: 10.1007/7854_2018_80. [Epub ahead of print]

**19.**

[Reduced lateral occipital gray matter volume is associated with physical frailty and cognitive impairment in Parkinson's disease.](https://www.ncbi.nlm.nih.gov/pubmed/30523452)

**Chen YS, Chen HL, Lu CH, Chen MH, Chou KH, Tsai NW, Yu CC, Chiang PL, Lin WC.**

**Eur Radiol. 2019 May;29(5):2659-2668. doi: 10.1007/s00330-018-5855-7. Epub 2018 Dec 6.**

**20.**

[Cognitive Frailty is Associated with Fall-Related Fracture among Older People.](https://www.ncbi.nlm.nih.gov/pubmed/30498829)

**Tsutsumimoto K, Doi T, Makizako H, Hotta R, Nakakubo S, Makino K, Suzuki T, Shimada H.**

**J Nutr Health Aging. 2018;22(10):1216-1220. doi: 10.1007/s12603-018-1131-4.**

21. [Excluded: Review]

[Healthy Aging: American Geriatrics Society White Paper Executive Summary.](https://www.ncbi.nlm.nih.gov/pubmed/30382585)

Friedman SM, Mulhausen P, Cleveland ML, Coll PP, Daniel KM, Hayward AD, Shah K, Skudlarska B, White HK.

J Am Geriatr Soc. 2019 Jan;67(1):17-20. doi: 10.1111/jgs.15644. Epub 2018 Nov 1.

22. [Excluded: Pathologies others than neurodegenerative disorders]

[Cancer-Related Cognitive Outcomes Among Older Breast Cancer Survivors in the Thinking and Living With Cancer Study.](https://www.ncbi.nlm.nih.gov/pubmed/30281396)

Mandelblatt JS, Small BJ, Luta G, Hurria A, Jim H, McDonald BC, Graham D, Zhou X, Clapp J, Zhai W, Breen E, Carroll JE, Denduluri N, Dilawari A, Extermann M, Isaacs C, Jacobsen PB, Kobayashi LC, Holohan Nudelman K, Root J, Stern RA, Tometich D, Turner R, VanMeter JW, Saykin AJ, Ahles T.

J Clin Oncol. 2018 Oct 3:JCO1800140. doi: 10.1200/JCO.18.00140. [Epub ahead of print]

**23.**

[**Effects of Resistance Exercise Training on Cognitive Function and Physical Performance in Cognitive Frailty: A Randomized Controlled Trial.**](https://www.ncbi.nlm.nih.gov/pubmed/30272098)

**Yoon DH, Lee JY, Song W.**

**J Nutr Health Aging. 2018;22(8):944-951. doi: 10.1007/s12603-018-1090-9.**

**24.**

[**Cognitive Frailty Predicts Incident Dementia among Community-Dwelling Older People.**](https://www.ncbi.nlm.nih.gov/pubmed/30200236)

**Shimada H, Doi T, Lee S, Makizako H, Chen LK, Arai H.**

**J Clin Med. 2018 Aug 30;7(9). pii: E250. doi: 10.3390/jcm7090250.**

25. [Excluded: Pathologies others than neurodegenerative disorders]

[Cognitive Performance and **Frailty** in Older HIV-Positive Adults.](https://www.ncbi.nlm.nih.gov/pubmed/29957675)

Paul RH, Cooley SA, Garcia-Egan PM, Ances BM.

J Acquir Immune Defic Syndr. 2018 Nov 1;79(3):375-380. doi: 10.1097/QAI.0000000000001790.

**26.**

[**Comparison of cognitive functions among frail and prefrail older adults: a clinical perspective.**](https://www.ncbi.nlm.nih.gov/pubmed/29954464)

**da Silva Alves BB, de Oliveira Barbosa E, de Moraes Pimentel D, Carneiro LSF, Rodrigues ACMA, Deslandes AC, Alves MR, Rodrigues VD, Pereira EL, de Paula AMB, Pupe CCB, Monteiro-Junior RS.**

**Int Psychogeriatr. 2019 Feb;31(2):297-301. doi: 10.1017/S1041610218000765. Epub 2018 Jun 29.**

**27.**

[**The role of social frailty in explaining the association between hearing problems and mild cognitive impairment in older adults.**](https://www.ncbi.nlm.nih.gov/pubmed/29890382)

**Bae S, Lee S, Lee S, Jung S, Makino K, Park H, Shimada H.**

**Arch Gerontol Geriatr. 2018 Sep - Oct;78:45-50. doi: 10.1016/j.archger.2018.05.025. Epub 2018 Jun 1.**

28. [Excluded: Pathologies others than neurodegenerative disorders]

[Physical **frailty** and cognitive **function** among men with cardiovascular disease.](https://www.ncbi.nlm.nih.gov/pubmed/29864738)

Weinstein G, Lutski M, Goldbourt U, Tanne D.

Arch Gerontol Geriatr. 2018 Sep - Oct;78:1-6. doi: 10.1016/j.archger.2018.05.013. Epub 2018 May 29.

29. [Excluded: Pathologies others than neurodegenerative disorders]

[Ability of postoperative delirium to predict intermediate-term postoperative cognitive **function** in patients undergoing elective surgery at an academic medical centre: protocol for a prospective cohort study.](https://www.ncbi.nlm.nih.gov/pubmed/29550773)

Aranake-Chrisinger A, Cheng JZ, Muench MR, Tang R, Mickle A, Maybrier H, Lin N, Wildes T, Lenze E, Avidan MS.

BMJ Open. 2018 Mar 17;8(3):e017079. doi: 10.1136/bmjopen-2017-017079.

**30.**

[**Cognitive Frailty and Incidence of Dementia in Older Persons.**](https://www.ncbi.nlm.nih.gov/pubmed/29405232)

**Shimada H, Makizako H, Tsutsumimoto K, Doi T, Lee S, Suzuki T.**

**J Prev Alzheimers Dis. 2018;5(1):42-48. doi: 10.14283/jpad.2017.29.**

31. [Excluded: Pathologies others than neurodegenerative disorders]

[Verbal fluency in elderly with and without hypertension and diabetes from the FIBRA study in Ermelino Matarazzo.](https://www.ncbi.nlm.nih.gov/pubmed/29354222)

Morelli NL, Cachioni M, Lopes A, Batistoni SST, Falcão DVDS, Neri AL, Yassuda MS.

Dement Neuropsychol. 2017 Oct-Dec;11(4):413-418. doi: 10.1590/1980-57642016dn11-040011.

32. [Excluded: Review]

[**Frailty** in elderly diabetes patients.](https://www.ncbi.nlm.nih.gov/pubmed/29238004)

Yanase T, Yanagita I, Muta K, Nawata H.

Endocr J. 2018 Jan 30;65(1):1-11. doi: 10.1507/endocrj.EJ17-0390. Epub 2017 Dec 14. Review.

**33.**

[**Neuropsychological Correlates of Pre-Frailty in Neurocognitive Disorders: A Possible Role for Metacognitive Dysfunction and Mood Changes.**](https://www.ncbi.nlm.nih.gov/pubmed/29188218)

**Amanzio M, Palermo S, Zucca M, Rosato R, Rubino E, Leotta D, Bartoli M, Rainero I.**

**Front Med (Lausanne). 2017 Nov 15;4:199. doi: 10.3389/fmed.2017.00199. eCollection 2017.**

**34.**

[**Global Performance of Executive Function Is Predictor of Risk of Frailty and Disability in Older Adults.**](https://www.ncbi.nlm.nih.gov/pubmed/29083438)

**Rosado-Artalejo C, Carnicero JA, Losa-Reyna J, Castillo C, Cobos-Antoranz B, Alfaro-Acha A, Rodríguez-Mañas L, García-García FJ.**

**J Nutr Health Aging. 2017;21(9):980-987. doi: 10.1007/s12603-017-0895-2.**

**35.**

[**Cognitive Change in Rehabilitation Patients with Dementia: Prevalence and Association with Rehabilitation Success.**](https://www.ncbi.nlm.nih.gov/pubmed/28984597)

**Dutzi I, Schwenk M, Kirchner M, Bauer JM, Hauer K.**

**J Alzheimers Dis. 2017;60(3):1171-1182. doi: 10.3233/JAD-170401.**

**36.**

[**Virtual Reality as a Potential Tool to Face Frailty Challenges.**](https://www.ncbi.nlm.nih.gov/pubmed/28928703)

**Serino S, Barello S, Miraglia F, Triberti S, Repetto C.**

**Front Psychol. 2017 Sep 5;8:1541. doi: 10.3389/fpsyg.2017.01541. eCollection 2017.**

37. [Excluded: Animal Model]

[Pre-existing weakness is critical for the occurrence of postoperative cognitive dysfunction in mice of the same age.](https://www.ncbi.nlm.nih.gov/pubmed/28787017)

Tang Y, Wang X, Zhang S, Duan S, Qing W, Chen G, Ye F, Le Y, Ouyang W.

PLoS One. 2017 Aug 7;12(8):e0182471. doi: 10.1371/journal.pone.0182471. eCollection 2017.

38. [Excluded: Pathologies others than neurodegenerative disorders]

[ANMCO/SIC/SICI-GISE/SICCH **Executive** Summary of Consensus Document on Risk Stratification in elderly patients with aortic stenosis before surgery or transcatheter aortic valve replacement.](https://www.ncbi.nlm.nih.gov/pubmed/28751850)

Pulignano G, Gulizia MM, Baldasseroni S, Bedogni F, Cioffi G, Indolfi C, Romeo F, Murrone A, Musumeci F, Parolari A, Patanè L, Pino PG, Mongiardo A, Spaccarotella C, Di Bartolomeo R, Musumeci G.

Eur Heart J Suppl. 2017 May;19(Suppl D):D354-D369. doi: 10.1093/eurheartj/sux012. Epub 2017 May 2.

**39.**

[**Association of Social Frailty With Both Cognitive and Physical Deficits Among Older People.**](https://www.ncbi.nlm.nih.gov/pubmed/28411094)

**Tsutsumimoto K, Doi T, Makizako H, Hotta R, Nakakubo S, Makino K, Suzuki T, Shimada H.**

**J Am Med Dir Assoc. 2017 Jul 1;18(7):603-607. doi: 10.1016/j.jamda.2017.02.004. Epub 2017 Apr 11.**

40. [Excluded: Study Protocol]

[The effect of an interactive cycling training on cognitive functioning in older adults with mild dementia: study protocol for a randomized controlled trial.](https://www.ncbi.nlm.nih.gov/pubmed/28327083)

Karssemeijer EG, Bossers WJ, Aaronson JA, Kessels RP, Olde Rikkert MG.

BMC Geriatr. 2017 Mar 21;17(1):73. doi: 10.1186/s12877-017-0464-x.

**41.**

[**Gait Speed and Grip Strength Reflect Cognitive Impairment and Are Modestly Related to Incident Cognitive Decline in Memory Clinic Patients With Subjective Cognitive Decline and Mild Cognitive Impairment: Findings From the 4C Study.**](https://www.ncbi.nlm.nih.gov/pubmed/28177065)

**Hooghiemstra AM, Ramakers IHGB, Sistermans N, Pijnenburg YAL, Aalten P, Hamel REG, Melis RJF, Verhey FRJ, Olde Rikkert MGM, Scheltens P, van der Flier WM; 4C Study Group.**

**J Gerontol A Biol Sci Med Sci. 2017 Jun 1;72(6):846-854. doi: 10.1093/gerona/glx003.**

42. [Excluded: Review]

[Assessing the Current State of Cognitive Frailty: Measurement Properties.](https://www.ncbi.nlm.nih.gov/pubmed/28112769)

Sargent L, Brown R.

J Nutr Health Aging. 2017;21(2):152-160. doi: 10.1007/s12603-016-0735-9. Review.

43. [Excluded: Pathologies others than neurodegenerative disorders]

[Lower **Frailty** Is Associated with Successful Cognitive Aging Among Older Adults with HIV.](https://www.ncbi.nlm.nih.gov/pubmed/27869500)

Wallace LM, Ferrara M, Brothers TD, Garlassi S, Kirkland SA, Theou O, Zona S, Mussini C, Moore D, Rockwood K, Guaraldi G.

AIDS Res Hum Retroviruses. 2017 Feb;33(2):157-163. doi: 10.1089/AID.2016.0189. Epub 2017 Jan 3.

44. [Excluded: Pathologies others than neurodegenerative disorders]

[Cognitive Impairment in Advanced Chronic Kidney Disease: The Canadian **Frailty** Observation and Interventions Trial.](https://www.ncbi.nlm.nih.gov/pubmed/27798938)

Foster R, Walker S, Brar R, Hiebert B, Komenda P, Rigatto C, Storsley L, Prasad B, Bohm C, Tangri N.

Am J Nephrol. 2016;44(6):473-480. Epub 2016 Nov 1.

45. [Excluded: Review]

[Knowledge Gaps in Cardiovascular Care of Older Adults: A Scientific Statement from the American Heart Association, American College of Cardiology, and American Geriatrics Society: **Executive** Summary.](https://www.ncbi.nlm.nih.gov/pubmed/27673575)

Rich MW, Chyun DA, Skolnick AH, Alexander KP, Forman DE, Kitzman DW, Maurer MS, McClurken JB, Resnick BM, Shen WK, Tirschwell DL.

J Am Geriatr Soc. 2016 Nov;64(11):2185-2192. doi: 10.1111/jgs.14576. Epub 2016 Sep 27. Review.

**46.**

[**The Early Indicators of Functional Decrease in Mild Cognitive Impairment.**](https://www.ncbi.nlm.nih.gov/pubmed/27570509)

**Kubicki A, Fautrelle L, Bourrelier J, Rouaud O, Mourey F.**

**Front Aging Neurosci. 2016 Aug 12;8:193. doi: 10.3389/fnagi.2016.00193. eCollection 2016.**

**47.**

[**Impact of Cognitive Frailty on Daily Activities in Older Persons.**](https://www.ncbi.nlm.nih.gov/pubmed/27499306)

**Shimada H, Makizako H, Lee S, Doi T, Lee S, Tsutsumimoto K, Harada K, Hotta R, Bae S, Nakakubo S, Harada K, Suzuki T.**

**J Nutr Health Aging. 2016;20(7):729-35. doi: 10.1007/s12603-016-0685-2.**

**48.**

[**Declines and Impairment in Executive Function Predict Onset of Physical Frailty.**](https://www.ncbi.nlm.nih.gov/pubmed/27084314)

**Gross AL, Xue QL, Bandeen-Roche K, Fried LP, Varadhan R, McAdams-DeMarco MA, Walston J, Carlson MC.**

**J Gerontol A Biol Sci Med Sci. 2016 Dec;71(12):1624-1630. Epub 2016 Apr 15.**

**49.**

[**Cognitive impairment is associated with the absence of fear of falling in community-dwelling frail older adults.**](https://www.ncbi.nlm.nih.gov/pubmed/26792588)

**Shirooka H, Nishiguchi S, Fukutani N, Tashiro Y, Nozaki Y, Hirata H, Yamaguchi M, Tasaka S, Matsushita T, Matsubara K, Aoyama T.**

**Geriatr Gerontol Int. 2017 Feb;17(2):232-238. doi: 10.1111/ggi.12702. Epub 2016 Jan 21.**

50. [Excluded: Pathologies others than neurodegenerative disorders]

[The relationship between neuropsychological assessment, numeracy, and functional status in older adults with type 1 diabetes.](https://www.ncbi.nlm.nih.gov/pubmed/26605669)

Chaytor NS, Riddlesworth TD, Bzdick S, Odegard PS, Gray SL, Lock JP, DuBose SN, Beck RW; T1D Exchange Severe Hypoglycemia in Older Adults with Type 1 Diabetes Study Group.

Neuropsychol Rehabil. 2017 Jun;27(4):507-521. doi: 10.1080/09602011.2015.1116448. Epub 2015 Nov 25.

**51.**

[**Stress Regulation as a Link between Executive Function and Pre-Frailty in Older Adults.**](https://www.ncbi.nlm.nih.gov/pubmed/26412287)

**Roiland RA, Lin F, Phelan C, Chapman BP.**

**J Nutr Health Aging. 2015 Oct;19(8):828-38. doi: 10.1007/s12603-015-0476-1.**

52. [Excluded: Pathologies others than neurodegenerative disorders]

[Physical **frailty** in late-life depression is associated with deficits in speed-dependent **executive functions**.](https://www.ncbi.nlm.nih.gov/pubmed/26313370)

Potter GG, McQuoid DR, Whitson HE, Steffens DC.

Int J Geriatr Psychiatry. 2016 May;31(5):466-74. doi: 10.1002/gps.4351. Epub 2015 Aug 27.

53. [Excluded: Review]

[**Frailty** and cognitive decline: how do they relate?](https://www.ncbi.nlm.nih.gov/pubmed/25405314)

Canevelli M, Cesari M, van Kan GA.

Curr Opin Clin Nutr Metab Care. 2015 Jan;18(1):43-50. doi: 10.1097/MCO.0000000000000133. Review.

**54.**

[**Cognitive function in the prefrailty and frailty syndrome.**](https://www.ncbi.nlm.nih.gov/pubmed/25370593)

**Robertson DA, Savva GM, Coen RF, Kenny RA.**

**J Am Geriatr Soc. 2014 Nov;62(11):2118-24. doi: 10.1111/jgs.13111. Epub 2014 Nov 4.**

**55.**

[**Reconceptualizing balance: attributes associated with balance performance.**](https://www.ncbi.nlm.nih.gov/pubmed/24952097)

**Thomas JC, Odonkor C, Griffith L, Holt N, Percac-Lima S, Leveille S, Ni P, Latham NK, Jette AM, Bean JF.**

**Exp Gerontol. 2014 Sep;57:218-23. doi: 10.1016/j.exger.2014.06.012. Epub 2014 Jun 18.**

**56.**

[**Linking cognition and frailty in middle and old age: metabolic syndrome matters.**](https://www.ncbi.nlm.nih.gov/pubmed/24733716)

**Lin F, Roiland R, Chen DG, Qiu C.**

**Int J Geriatr Psychiatry. 2015 Jan;30(1):64-71. doi: 10.1002/gps.4115. Epub 2014 Apr 15.**

57. [Excluded: Pathologies others than neurodegenerative disorders]

[Pilot study of younger and older HIV-infected adults using traditional and novel functional assessments.](https://www.ncbi.nlm.nih.gov/pubmed/23924589)

Sandkovsky U, Robertson KR, Meza JL, High RR, Bonasera SJ, Fisher CM, Marsh AJ, Sheehy MK, Fox HS, Swindells S.

HIV Clin Trials. 2013 Jul-Aug;14(4):165-74. doi: 10.1310/hct1404-165.

**58.**

[**Serum 25-hydroxyvitamin D is associated with cognitive executive function in Dutch prefrail and frail elderly: a cross-sectional study exploring the associations of 25-hydroxyvitamin D with glucose metabolism, cognitive performance and depression.**](https://www.ncbi.nlm.nih.gov/pubmed/23921196)

**Brouwer-Brolsma EM, van de Rest O, Tieland M, van der Zwaluw NL, Steegenga WT, Adam JJ, van Loon LJ, Feskens EJ, de Groot LC.**

**J Am Med Dir Assoc. 2013 Nov;14(11):852.e9-17. doi: 10.1016/j.jamda.2013.06.010. Epub 2013 Aug 3.**

**59.**

[**Experience Corps: a dual trial to promote the health of older adults and children's academic success.**](https://www.ncbi.nlm.nih.gov/pubmed/23680986)

**Fried LP, Carlson MC, McGill S, Seeman T, Xue QL, Frick K, Tan E, Tanner EK, Barron J, Frangakis C, Piferi R, Martinez I, Gruenewald T, Martin BK, Berry-Vaughn L, Stewart J, Dickersin K, Willging PR, Rebok GW.**

**Contemp Clin Trials. 2013 Sep;36(1):1-13. doi: 10.1016/j.cct.2013.05.003. Epub 2013 May 13.**

**60.**

[**Combined prevalence of frailty and mild cognitive impairment in a population of elderly Japanese people.**](https://www.ncbi.nlm.nih.gov/pubmed/23669054)

**Shimada H, Makizako H, Doi T, Yoshida D, Tsutsumimoto K, Anan Y, Uemura K, Ito T, Lee S, Park H, Suzuki T.**

**J Am Med Dir Assoc. 2013 Jul;14(7):518-24. doi: 10.1016/j.jamda.2013.03.010. Epub 2013 May 10.**

61. [Excluded: Other Topic]

[Leisure activity, health, and medical correlates of neurocognitive performance among monozygotic twins: the Older Australian Twins Study.](https://www.ncbi.nlm.nih.gov/pubmed/23668997)

Lee T, Lipnicki DM, Crawford JD, Henry JD, Trollor JN, Ames D, Wright MJ, Sachdev PS; OATS Research Team.

J Gerontol B Psychol Sci Soc Sci. 2014 Jul;69(4):514-22. doi: 10.1093/geronb/gbt031. Epub 2013 May 13.

**62.**

[**Sustained attention and frailty in the older adult population.**](https://www.ncbi.nlm.nih.gov/pubmed/23525545)

**O'Halloran AM, Finucane C, Savva GM, Robertson IH, Kenny RA.**

**J Gerontol B Psychol Sci Soc Sci. 2014 Mar;69(2):147-56. doi: 10.1093/geronb/gbt009. Epub 2013 Mar 22.**

**63.**

[**Benefits of physical exercise training on cognition and quality of life in frail older adults.**](https://www.ncbi.nlm.nih.gov/pubmed/22929394)

**Langlois F, Vu TT, Chassé K, Dupuis G, Kergoat MJ, Bherer L.**

**J Gerontol B Psychol Sci Soc Sci. 2013 May;68(3):400-4. doi: 10.1093/geronb/gbs069. Epub 2012 Aug 28.**

**64.**

[**The multiple dimensions of frailty: physical capacity, cognition, and quality of life.**](https://www.ncbi.nlm.nih.gov/pubmed/22717010)

**Langlois F, Vu TT, Kergoat MJ, Chassé K, Dupuis G, Bherer L.**

**Int Psychogeriatr. 2012 Sep;24(9):1429-36. doi: 10.1017/S1041610212000634. Epub 2012 Apr 25.**

**65.**

[**Apathy as marker of frail status.**](https://www.ncbi.nlm.nih.gov/pubmed/22500230)

**Semprini R, Lubrano A, Misaggi G, Martorana A.**

**J Aging Res. 2012;2012:436251. doi: 10.1155/2012/436251. Epub 2012 Feb 12.**

**66.**

[**Geriatric syndromes in older homeless adults.**](https://www.ncbi.nlm.nih.gov/pubmed/21879368)

**Brown RT, Kiely DK, Bharel M, Mitchell SL.**

**J Gen Intern Med. 2012 Jan;27(1):16-22. doi: 10.1007/s11606-011-1848-9. Epub 2011 Aug 31.**

67. [Excluded: Review]

[Developing novel therapeutic approaches to **frailty**.](https://www.ncbi.nlm.nih.gov/pubmed/19860686)

Morley JE.

Curr Pharm Des. 2009;15(29):3384-95. Review.

**68.**

[**Validation and comparison of two frailty indexes: The MOBILIZE Boston Study.**](https://www.ncbi.nlm.nih.gov/pubmed/19682112)

**Kiely DK, Cupples LA, Lipsitz LA.**

**J Am Geriatr Soc. 2009 Sep;57(9):1532-9. doi: 10.1111/j.1532-5415.2009.02394.x. Epub 2009 Jul 21.**

69. [Excluded: Review]

[Diabetes, sarcopenia, and **frailty**.](https://www.ncbi.nlm.nih.gov/pubmed/18672182)

Morley JE.

Clin Geriatr Med. 2008 Aug;24(3):455-69, vi. doi: 10.1016/j.cger.2008.03.004. Review.
